# Practicing COVID-19 Public Health Measures Is Associated With Anxiety and Depression in Undergraduate University Students

**DOI:** 10.3389/fpubh.2022.941730

**Published:** 2022-07-06

**Authors:** Kelley Holladay, David Lardier, Fabiano T. Amorim, Micah Zuhl, Kathryn E. Coakley

**Affiliations:** ^1^Brooks Rehabilitation College of Healthcare Sciences, Jacksonville University, Jacksonville, FL, United States; ^2^The Department of Psychiatry and Behavioral Sciences, The University of New Mexico School of Medicine, The University of New Mexico, Albuquerque, NM, United States; ^3^Department of Health, Exercise and Sports Sciences, The University of New Mexico, Albuquerque, NM, United States; ^4^School Health Sciences, Central Michigan University, Mount Pleasant, MI, United States; ^5^Department of Individual, Family and Community Education, University of New Mexico, Albuquerque, NM, United States

**Keywords:** mental health, depression, anxiety, undergraduate, COVID-19

## Abstract

**Background:**

COVID-19 has affected mental health globally, increasing depression and anxiety. This study examined relationships between practicing COVID-19-related public health measures and depression and anxiety in young adult students.

**Methods:**

A sample of 755 undergraduate students 18–25 years of age at a large public university completed a cross-sectional survey in fall 2020 during the pandemic (response rate = 18.9%). The survey included demographic questions, anxiety and depression screeners (GAD-7 and PHQ-9), and questions on practicing public health measures (stay-at-home orders, quarantining, social distancing, etc.) since March 2020. Multivariate logistic regression was utilized to calculated adjusted odds between practicing public health measures and anxiety and depression.

**Results:**

The majority of respondents reported practicing public health measures; however, 53% experienced anxiety (GAD-7 score >10) and 57% experienced depression (PHQ-9 score >10) in the 2 weeks prior to completing the survey. Participants who quarantined had significantly higher odds of anxiety (AOR = 1.44; 95% CL 1.07, 1.96) and depression (AOR = 1.77; 95% CL 1.30, 2.41) than those who did not. Participants who self-isolated also had significantly higher odds of anxiety (AOR = 1.53; 95% CL 1.13, 2.08) and depression (AOR = 1.87; 95% CL 1.37, 2.56) compared to those who did not. Moving/changing living situations in response to the pandemic also increased odds of depression (AOR = 1.86; 95% CL 1.33, 2.60).

**Conclusion:**

Young adult undergraduate students experienced a high prevalence of anxiety and depression. Quarantining, self-isolating, and moving/changing living situations increased odds of anxiety and/or depression. The public health measures necessary for COVID-19 control and prevention may adversely affect mental health.

## Introduction

In January 2020, the World Health Organization (WHO) declared COVID-19, the disease caused by the novel coronavirus SARS-CoV2, a public health emergency of international concern (1). The first case of the novel coronavirus was reported in the United States between December 2019–January 2020 (2, 3). It was not until March 19, however, that public health measures were implemented by individual states including stay-at-home orders, social distancing, and wearing masks or face coverings to control spread and to limit overwhelming hospital systems (4). In addition, quarantine and self-isolation periods were implemented under state-specific public health orders for individuals meeting criteria based on exposure and/or display of Covid-19 symptoms, like fever or chills (4). As COVID-19 spread through communities across the United States, institutions of higher education closed campuses and moved to remote learning modalities, displacing thousands of students (5).

The COVID-19 pandemic has had a global impact on mental health and well-ness (5). Evidence suggests adults under 45 years of age have been the most psychologically affected by lockdown measures, particularly due to loneliness and vulnerability to stress, financial distress, and sleep disturbances; (6) however, few studies have examined university students or young adults. Prior to the pandemic an estimated 41.6% of college students suffered from anxiety, and 36.4% were affected by depression (7). As of June 2020, the Centers for Disease Control and Prevention (CDC) reported that 75% of young adults 18–24 years of age suffered from at least one mental or behavioral health symptom, and more than 25% had seriously contemplated suicide in the prior month (8). Other studies have also shown increased anxiety, depression, and suicidal ideation in college students due to the pandemic (9, 10).

In university students, anxieties related to the pandemic may be exacerbated by practicing necessary public health measures and by changes in higher education such as closed campuses and online learning with limited face-to-face interaction with professors or peers (11). Extended quarantine and isolation periods are associated with psychological distress and other mental health-related concerns including decreased concentration, disrupted sleep patterns, feelings of unhappiness, loss of purpose, and substance use (12). In young adults, social support through peer networks is important and social isolation and loneliness due to the pandemic may impact mental health (13). Information on the relationship between practicing public health measures and mental health in undergraduate students is limited. The impact of public health measures on undergraduate students mental health is important to examine as colleges and universities continue to offer remote delivery courses.

This study explored relationships between practicing public health measures and anxiety and depression in a sample of undergraduate students 18–25 years of age at a large public university. We hypothesized that practicing public health measures including social distancing, quarantining, self-isolation, and discontinuing to see family and friends would increase of anxiety and depressive symptomology.

## Methods

### Participants and Recruitment

This investigation examined how the COVID-19 pandemic that necessitated public health measures, like quarantining and self-isolating, influenced anxiety and depression and undergraduate University students. As such, undergraduate students 18–25 years of age enrolled at a large public university in the southwest United States in spring and fall 2020 were eligible for inclusion in this study. Students were recruited for 2 weeks in fall 2020 during a second wave of the COVID-19 pandemic. The study recruitment email was sent to 4,000 students, randomly selected to create a representative sample of the university's race/ethnicity and gender composition, Pell grant eligibility (an indicator of low-income status), and campus enrollment (at the main campus or at one of the university's branch campuses). Participants provided consent by starting the survey. The study was approved by the university's Institutional Review Board and conformed to principles embodied in the Declaration of Helsinki. Results will be disseminated to participants through the university.

### Instruments

Data were collected *via* Opinio, a secure online survey tool used by the university for research (14). The survey included screening questions assessing the potential participants' age and student status. The survey then included demographic questions (age, race/ethnicity, gender, sexuality, academic standing, Pell grant eligibility, employment); the seven-item Generalized Anxiety Disorder Scale 7 (GAD-7), (15) a validated measure of anxiety in the past 2 weeks; the Patient Health Questionnaire 9 (PHQ-9), (16) a validated measure of depression in the past 2 weeks; and questions on COVID-19 testing and practicing public health measures (i.e., stay at home orders, social distancing, wearing a mask or face covering) since the start of the pandemic in March 2020.

### Data Analysis

Responses to survey questions were not required; however, only participants who responded to questions on public health measures were included in data analysis. All data analyses were performed using Statistical Analysis Software (SAS) Version 9.4. Demographic data are presented descriptively (mean, standard deviation, frequency, percentage). The GAD-7 and PHQ-9 were scored according to standard guidelines. Means and standard deviations (SD) were calculated for each instrument for the full sample and by demographic subgroups. Raw GAD-7 scores were dichotomized to anxiety (score >10) or no anxiety (score <10) (15, 17). Raw PHQ-9 scores were dichotomized to depression (score >10) or no depression (score <10) (16). The prevalence of anxiety and depression were calculated for the full sample.

Participants also indicated if seven public health measures applied to them during the COVID-19 pandemic, since March 2020. The following measures were assessed dichotomously (“no” = 0 or “yes” = 1): staying at home due to stay at home orders, quarantining, self-isolating, practicing social distancing (staying at least six feet from others), wearing a mask/face covering, moving/changing living situation, continuing to see family and friends, or making no changes.

The frequency and percent of participants reporting anxiety (GAD-7 score >10) and depression (PHQ-9 score >10) were calculated for those reporting practicing vs. not practicing each public health measure. Differences in anxiety and depression as binary variables (present or not present) were assessed using Fisher's exact tests (*P* <0.05 indicated statistical significance). All demographic variables (age, gender, race/ethnicity, sexuality, employment status, and Pell grant eligibility) were considered candidates for inclusion as Co-variates in multivariate models. To determine Co-variates, raw GAD-7 and PHQ-9 scores were compared between demographic subgroups utilizing analysis of variance (ANOVA). A *P*-value <0.20 was used to determine Co-variates for inclusion in logistic regression analyses, opposed to *P* < 0.05 because traditional levels of *P* < 0.05 can fail in identifying variables known to be important (18).

Multivariate logistic regression was utilized to calculate adjusted odds ratios (AOR) between practicing public health measures and anxiety (GAD-7 score >10) and depression (PHQ-9 score >10) as separate outcomes. 95% Wald Confidence Limits were also calculated for AORs.

## Results

Recruitment emails were sent to 4,000 randomly selected students in October 2020. Eight-hundred fifty-two individuals started the survey. Of those, 97 were excluded (23 did not meet inclusion criteria, five reported an age outside the age range, and 69 did not answer questions regarding public health measures). The final sample included 755 participants, yielding a response rate of 18.9%.

The average age of participants was 21.3 ± 1.6 years. Shown in Table 1, the majority were 21–25 years of age (*n* = 499; 66.4%); female (*n* = 461; 61.4%); and White (*n* = 239; 31.7%) or Hispanic, Latino, or Spanish (*n* = 218; 28.9%). The majority reported heterosexual (*n* = 519; 69.0%) or bisexual (*n* = 84; 11.2%) sexual orientation. Sixty-one percent were employed at the time of taking the survey, either full-time or part-time. Over half of participants were eligible for Pell grants (*n* = 377; 50.1%), an indicator of low-income status. Most participants reported they had not been tested for COVID-19 since March 2020 (*n* = 470; 62.3%). Of those that had been tested (*n* = 285; 37.7%), 94% (*n* = 267) tested negative.

**Table 1:** Demographic characteristics of study participants and raw GAD-7 and PHQ-9 scores.

**Demographics**	***n***	**%**	**GAD-7**		**PHQ-9**	
			**Mean (SD)**	***P*-value^**a**^**	**Mean (SD)**	***P*-value^**a**^**
**Age (years)**						
18–20	252	33.6	10.1 (6.2)	0.28	10.9 (6.9)	0.54
21–25	499	66.4	10.6 (6.1)		11.2 (7.0)	
**Gender**						
Male	261	34.8	8.9 (6.2)	<0.01	9.6 (7.0)	<0.01
Female	461	61.4	11.1 (6.0)		11.6 (6.9)	
Transgender, gender fluid, other	29	3.9	14.1 (4.5)		15.4 (5.4)	
**Race/Ethnicity**						
American Indian/Alaskan Native	55	7.3	9.2 (6.9)	0.27	10.1 (8.2)	0.51
Asian	56	7.4	9.1 (7.3)		9.6 (7.5)	
Black or African American	11	1.5	11.5 (6.1)		11.3 (7.2)	
Hispanic, Latino, or Spanish	218	28.9	10.7 (6.0)		11.2 (6.9)	
White	239	31.7	10.8 (6.0)		11.4 (7.0)	
Other/>1 race	175	23.2	10.3 (5.9)		11.3 (6.5)	
**Sexual orientation**						
Heterosexual	519	69.0	9.5 (6.2)	<0.01	10.1 (7.0)	<0.01
Gay, lesbian, queer	53	7.1	11.7 (5.7)		12.1 (7.4)	
Bisexual	84	11.2	13.5 (5.0)		14.5 (5.9)	
Asexual	34	4.5	9.5 (6.6)		10.4 (6.5)	
Other/questioning	62	8.2	13.5 (4.8)		14.0 (5.7)	
**Pell grant eligibility**						
No	323	42.9	10.1 (6.2)	0.54	10.4 (6.8)	0.07
Yes	377	50.1	10.7 (6.0)		11.6 (7.0)	
I'm not sure	53	7.0	10.5 (6.5)		11.5 (7.6)	
**Employment**						
Employed, full-time	111	14.7	11.3 (6.1)	0.16	11.7 (7.3)	0.10
Employed, part-time	349	46.4	10.0 (6.0)		10.8 (6.7)	
Not employed, looking for work	140	18.6	11.1 (6.2)		12.1 (6.9)	
Not employed, not looking for work	131	17.4	9.8 (6.4)		10.0 (7.2)	
Other	22	2.9	11.0 (5.9)		11.3 (6.9)	

*^a^Analysis of variance (ANOVA)*.

### Mental Health

Mean GAD-7 and PHQ-9 scores are reported in Table 1 across demographic subgroups. There were significant differences in mean GAD-7 and PHQ-9 scores by gender and sexuality (*P* <0.01). In the full sample, 53% (*n* = 400) were experiencing anxiety in the past 2 weeks based on a GAD-7 score of 10 or more. Mean GAD-7 score was 10.4 ± 6.1. In addition, 57% (*n* = 430) were experiencing depression in the past 2 weeks, based on a PHQ-9 score of 10 or more. Mean PHQ-9 score was 11.1 ± 7.0. Nearly two-thirds (*n* = 487; 64.5%) experienced depression or anxiety and nearly half (*n* = 343; 45.4%) were experiencing both anxiety and depression. Seventy-nine participants (10.5%) reported serious thoughts about ending their life in the past month.

### Practicing Public Health Measures

Prevalence of practicing public health measures are presented in Table 2. The majority of participants reported practicing the following public health measures since the start of the COVID-19 pandemic in March 2020: wearing a mask/face covering (*n* = 719; 95.2%), social distancing (*n* = 693; 91.8%), staying at home due to stay at home orders (*n* = 662; 87.7%), and quarantining (*n* = 404; 53.5%). The majority also reported continuing to see family and friends (*n* = 390; 51.7%). Fewer than half reported self-isolating (*n* = 359; 47.6%). One-third of the sample reported moving or changing living situations due to the pandemic (*n* = 254; 33.6%). Just 1.5% of the sample reported making no changes.

**Table 2:** Practicing public health measures and prevalence of anxiety and depression.

**Public health measures**	***n***	**%**	**Anxiety (GAD-7 score** **>****10)**			**Depression (PHQ-9 score** **>****10)**		
			***n***	**%**	***P*-value^**a**^**	***n***	**%**	***P*-value^**a**^**
**Stayed home due to stay at home orders**					0.51			0.82
No	93	12.3	46	49.5		52	55.9	
Yes	662	87.7	354	53.5		378	57.2	
**Quarantined**						<0.01		<0.01
No	351	46.5	165	47.0		172	49.0	
Yes	404	53.5	235	58.2		258	64.0	
**Self-isolated**					<0.01			<0.01
No	396	52.5	186	47.0		194	49.0	
Yes	359	47.6	214	59.6		236	65.9	
**Practiced social distancing**					0.79			0.28
No	62	8.2	34	54.8		31	50.0	
Yes	693	91.8	366	52.8		399	57.7	
**Wore a mask/face covering**					0.50			0.04
No	36	4.8	17	47.2		14	38.9	
Yes	719	95.2	383	53.3		416	57.9	
**Moved/changed living situations**					0.01			<0.01
No	501	66.4	249	49.7		259	51.8	
Yes	254	33.6	151	59.5		171	67.3	
**Continued to see family and friends**					0.049			0.46
No	365	48.3	207	56.7		213	58.5	
Yes	390	51.7	193	49.5		217	55.6	
**Made no changes**					0.55			1.00
No	744	98.5	393	52.8		424	57.1	
Yes	11	1.5	7	63.6		6	54.6	

*^a^Fisher's exact tests; P-value <0.05 indicates significance*.

### Public Health Measures and Mental Health

Table 2 also shows the prevalence of anxiety (GAD-7 score >10) and depression (PHQ-9 score >10) by practicing public health measures. Those who quarantined had a significantly higher prevalence of anxiety (58.2%) and depression (64.0%) compared to those who did not quarantine (47.0% and 49.0%; *P* < 0.01). Those who reported self-isolating also had a significantly higher prevalence of anxiety (59.6%) and depression (65.9%) compared to those who did not (47.0% and 49.0%; *P* < 0.01). Students who moved also had a significantly higher prevalence of anxiety (59.5%) and depression (67.3%) compared to those who did not (49.7% and 51.8%; *P* = 0.01 and *P* < 0.01, respectively). The prevalence of depression was also significantly higher in those that reported wearing a mask/face covering (*P* = 0.04), and the prevalence of anxiety was significantly higher in those that continued to see family and friends (*P* = 0.049).

In Co-variate analyses, GAD-7 scores differed by gender, sexuality, and employment (*P* < 0.20) while PHQ-9 scores differed by gender, Pell eligibility (a proxy of low-income), sexuality, and employment (*P* < 0.20). Associations between practicing public health measures and depression and anxiety, adjusting for these categorical variables are shown in Table 3.

**Table 3:** Practicing public health measures and adjusted odds of anxiety and depression.

Public health measures*	**Anxiety** **(GAD-7** **>****10)**			**Depression** **(PHQ-9** **>****10)**		
	**AOR^**a**^**	**95% CL**		**AOR^**b**^**	**95% CL**	
**Stayed home due to stay at home orders**	**1.00**	**0.63**	**1.59**	**0.95**	**0.59**	**1.51**
Quarantined	1.44	1.07	1.96	1.77	1.30	2.41
Self-isolated	1.53	1.13	2.08	1.87	1.37	2.56
Social distancing	0.79	0.46	1.35	1.15	0.66	1.98
Mask/face covering	1.09	0.54	2.20	1.93	0.93	3.98
Moved-changed living situations	1.35	0.98	1.87	1.86	1.33	2.60
Continued to see family and friends**	1.25	0.92	1.69	1.01	0.75	1.38

*Reference group = response of “no” except for^**^; ^a^Adjusted for gender, sexuality, employment status; ^b^Adjusted for gender, sexuality, Pell grant eligibility, employment status; CL, confidence limits.

Participants who quarantined had significantly higher odds of anxiety compared to those who did not (AOR = 1.44; 95% CL 1.07, 1.96). Participants who self-isolated also had significantly higher odds of anxiety compared to those who did not (AOR = 1.53; 95% CL 1.13, 2.08). Participants who quarantined had significantly higher odds of depression compared to those who did not (AOR = 1.77; 95% CL 1.30, 2.41). Those who self-isolated also had significantly higher odds of depression compared to those who did not (AOR = 1.87; 95% CL 1.37, 2.56). Finally, participants who reported moving or changing living situations in response to the COVID-19 pandemic, since March 2020, had significantly higher odds of depression compared to those who did not (AOR = 1.86; 95% CL 1.33, 2.60). Stay at home orders, practicing social distancing, wearing a mask/face covering, or continuing to see friends and family did not significantly increase or decrease odds of anxiety or depression.

## Discussion

Young adults and undergraduate students were particularly at risk for mental health distress during COVID-19 (5, 8–10, 19). Research exploring impacts of following public health measures (e.g., stay at home orders, social distancing, wearing a mask or face covering) on mental health during the COVID-19 pandemic is limited (12, 19–21). Extant research conducted earlier in the pandemic suggests anxiety and depression increased in university students due to COVID-19 (9). This study suggests a high prevalence of anxiety and depression in students and significant associations between practicing public health measures and mental health.

In the United States, the CDC advised institutions of higher education to apply public health measures ranging from remote educational delivery to closing campuses (22). In this sample of undergraduate students 18–25 years of age, over half experienced anxiety or depression in the past 2 weeks (53 and 57%, respectively), 45% experienced both, and 10% reported serious suicidal ideations in the past month. Those who reported practicing certain public health measures had a significantly higher prevalence of depression. For example, since March 2020, 64% of those who reported quarantining, 66% of those who reported self-isolating, and 67% of those who moved or changed living situations experienced depression based on PHQ-9 scores. Participants who reported quarantining, self-isolating, or moving or changing living situations had higher odds of anxiety and/or depression. Those who quarantined had 1.4 times the odds of anxiety and nearly 1.8 times the odds of depression compared to those who did not quarantine. Participants who reported self-isolating since March 2020 had 1.5 times the odds of anxiety and nearly 1.9 times the odds of depression compared to those who did not self-isolate. Moving or changing living situations also increased the odds of depression nearly 1.9 times. Prior research also suggests public health quarantines increase psychological distress like depression, with mental health distress increasing with quarantine length (20, 21).

Findings from this study are similar to recently published studies (12, 19–21). In June 2020, the CDC reported 49.1% of young adults 18–24 years of age had anxiety disorder, 52.3% had depressive disorder, and 62.9% had anxiety or depressive disorder (8). This study, conducted in October-November 2020, shows a similar and even slightly higher prevalence with 64.5% experiencing anxiety or depression. In this sample, 10.5% reported serious thoughts about ending their life in the past month, a lower estimate than CDC's report of 25.5% in young adults 18–24 years of age (8). Because suicide is the second leading cause of death among young adults, (23) there is a critical need to assess suicidal thoughts and implement preventive measures in young adults during the pandemic, given the increase in anxiety, depression, and other mental health concerns.

Academic performance was not captured in the current study and is a possible co-factor in the linkage between mental health outcomes and COVID-19 public health measures (24). Specifically, the alterations in study behaviors (e.g., studying in isolation vs. in a social gathering) and delivery of course content (e.g., shift from in-person instruction to online formats) due to lockdown measures increased likelihood of poor academic performance and thus mental health decline (or vice versa) (24). Moreover, students who reported lower academic concerns and levels of anxiety and depression were less likely to dropout during the COVID-19 pandemic (25, 26). We were unable to measure the impact of academic performance on reported outcomes, and is thus one limitation to the study.

### Limitations

Limitations of this study include the cross-sectional design employed. While this approach cannot track long-term effects of COVID-19 associated public health measures on undergraduate students' mental health, it offers necessary data to target current mental health of young adults. Generalizability is also limited. The sample size was large enough to produce significant results, however findings from undergraduate students 18–25 years cannot be generalized across age groups. Last, while demographics were limited, the sample mirrored the ethnic diversity of the university (23% identified as more than one race).

### Implications

The COVID-19 pandemic has adversely affected university students' mental health (27). Over half of participants experienced anxiety or depression in the past 2 weeks and 10% reported serious suicidal ideations in the past month. Quarantining, self-isolating and changing living situations significantly increase the odds of anxiety and/or depression. These results are critical to universities to ensure mental health services are available to students including telehealth and crisis counseling. Colleges and universities must continue mental health outreach and services to college students beyond this international pandemic (27, 28).

## Data Availability Statement

The raw data supporting the conclusions of this article will be made available by the authors, without undue reservation.

## Ethics Statement

The studies involving human participants were reviewed and approved by University of New Mexico Institutional Review Board. The patients/participants provided their written informed consent to participate in this study.

## Author Contributions

KH completed the paper. DL and KC completed data analysis. FA and MZ contributed to paper formation and critical analysis of the data. All authors collectively contributed to IRB submission, data collections, paper write up, and approved the submitted version.

## Conflict of Interest

The authors declare that the research was conducted in the absence of any commercial or financial relationships that could be construed as a potential conflict of interest.

## Publisher's Note

All claims expressed in this article are solely those of the authors and do not necessarily represent those of their affiliated organizations, or those of the publisher, the editors and the reviewers. Any product that may be evaluated in this article, or claim that may be made by its manufacturer, is not guaranteed or endorsed by the publisher.
